# The Subgingival Microbial Composition in Health and Periodontitis with Different Probing Depths

**DOI:** 10.3390/microorganisms13040930

**Published:** 2025-04-17

**Authors:** Jingyan Wang, Yiran Geng, Jing Guo, Jiahan Peng, He Xu, Bingqian Zhao, Shiyan Huang, Man Qin, Wenbin Du, Jing Tian

**Affiliations:** 1 Department of Pediatric Dentistry, Peking University School and Hospital of Stomatology, Beijing 100081, China; jenkins_1211@163.com (J.W.); pjhannah613@163.com (J.P.); kqxuhe2004@126.com (H.X.); zhaobingqian@pku.edu.cn (B.Z.); huangsy0404@163.com (S.H.); qin-man@foxmail.com (M.Q.); 2 National Center for Stomatology & National Clinical Research Center for Oral Diseases & National Engineering Research Center of Oral Biomaterials and Digital Medical Devices, Beijing 100081, China; gyr_1107@sina.com (Y.G.); 1710303114@pku.edu.cn (J.G.); 3 Department of Periodontology, Peking University School and Hospital of Stomatology, Beijing 100081, China; 4 State Key Laboratory of Microbial Diversity and Innovative Utilization, Institute of Microbiology, Chinese Academy of Sciences, Beijing 100101, China

**Keywords:** periodontitis, probing depth (PD), subgingival dental plaque, microbiome

## Abstract

The differences in microbiota between periodontitis and health have been extensively studied; however, knowledge about how the microbiota shifts from shallow to deep periodontal pockets remains limited despite its clinical importance in disease progres-sion and management. Patients diagnosed with stage III periodontitis commonly pre-sent varied probing depths (PD) within the same oral cavity, reflecting localized disease severity. This study aims to analyze the microbiome of subgingival plaques at various PDs in periodontitis patients. Subgingival plaques were collected from sixteen healthy subjects (health group) and periodontal pockets of sixteen stage III periodontitis pa-tients (PD 0–3 mm, PD 4–5 mm and PD 6–9 mm groups). A total of 64 subgingival plaque samples underwent 16S rRNA gene sequencing. The PD 6–9 mm group exhib-ited significantly higher alpha diversity than the health group, and distinct subgingival microbial community structures were observed in periodontitis patients, regardless of probing depth. The relative abundance of specific genera differed notably between health and periodontitis states; Corynebacterium and Cardiobacterium decreased, whereas Schaalia increased in shallow pockets (PD 0–3 mm) of periodontitis relative to the health group. Co-occurrence network analysis on the species level revealed that the PD 4–5 mm group had the most complex interspecies interactions, followed by the PD 6–9 mm and PD 0–3 mm groups. These findings indicate significant variations in mi-crobial diversity, composition, and interspecies interactions associated with periodon-tal health and periodontitis severity, highlighting their potential relevance for clinical diagnosis and targeted therapeutic strategies.

## 1. Introduction

Periodontitis is a prevalent and chronic infectious disease characterized by the loss of periodontal attachment, the resorption of alveolar bone, and the formation of periodontal pockets [1,2,3]. The primary etiological factor that triggers periodontitis is subgingival dental plaques, which are characterized by the formation of subgingival calculus and changes in microbial community composition [4,5,6]. Research has shown that alterations in the subgingival microbial ecological structure in patients with periodontitis result from potential pathogens with low-abundance becoming dominant rather than the disappearance of health-associated community members, indicating ecological succession [7,8]. In recent years, advancements in molecular biology techniques, such as Next-Generation Sequencing (NGS) of 16S rRNA genes, have enabled the accumulation of enormous sequencing data related to the comparison between health, gingivitis, and periodontitis [7,9,10]. Pathogens that were related to periodontitis have been well studied and summarized as complexes, such as Socransky’s complexes [11] and the recently proposed GF-MoR Complexes [12]. However, understanding microbial shifts across varying probing depths (PD), which reflect changes in ecological parameters such as oxygen tension [13], temperature [14], and pH [15], remains limited. Microbial differences between shallow and deep periodontal pockets likely reflect ongoing microbial succession in response to continuously evolving microenvironmental conditions within the pockets’ ecological niche [16].

Previous studies investigating microbiota at different probing depths have shown increased proportions of Spirochaetes, Bacteroidetes, Saccharibacteria (TM7), and Fusobacteria, with decreased Proteobacteria and Actinobacteria as pocket depth in-creases [17]. Other research reported linear correlations between specific bacterial abundances and probing depths, albeit with small sample sizes [18,19]. A recent study employing paper point sampling revealed similar microbial community structures in shallow sites (PD ≤ 3 mm) across health, stage I/II, and stage III/IV periodontitis sam-ples; significant differences emerged at moderate (PD 4–6 mm) and deep sites (PD ≥ 7 mm), including reduced Actinomyces and increased Treponema in deeper sites [20]. However, paper point sampling primarily reflects the out-layer of the subgingival plaque, potentially missing differences present in deeper subgingival plaque layers [21].

This study explored subgingival plaque microbiota in healthy individuals, health sites (PD 0–3 mm) of periodontitis patients, and diseased sites (PD 4–5 mm and PD 6–9 mm) of periodontitis patients using the curette collection method and 16S rRNA gene sequencing. The results demonstrated that the healthy individuals, health sites of periodontitis, and diseased sites of periodontitis showed significantly different microbiota diversity, structure, composition, and interspecies interactions.

## 2. Materials and Methods

### 2.1. Study Population and Clinical Examination

This study was conducted in accordance with the Declaration of Helsinki and approved by the Biomedical Ethics Committee of Peking University Hospital of Stomatology (PKUSSIRB-2024100084). Informed consent was obtained from all subjects involved in this study.

In 2024, sixteen periodontal healthy subjects and sixteen patients diagnosed with stage III periodontitis were enrolled at Peking University Hospital of Stomatology ac-cording to the 2018 International Classification of Periodontitis [22]. Healthy subjects had complete or reduced periodontal tissue, with bleeding on probing (BOP) sites less than 10% and probing depths (PD) ≤3 mm at all sites. Inclusion criteria were age be-tween 25 to 65 years, general good health, and at least 20 natural teeth. For the periodontitis group, each patient was required to have at least two teeth per quadrant (upper right, upper left, lower right, and lower left) with PD greater than 6 mm. Exclusion criteria included subgingival scaling experience or orthodontic appliance usage within the previous six months, presence of dental caries or mucosal diseases, antibiotics use within the last three months, smoking, pregnancy, or breastfeeding.

Probing depths were recorded using six sites per tooth. Subgingival plaque samples were categorized into four groups: health group (samples from healthy subjects), PD 0–3 mm, PD 4–5 mm, and PD 6–9 mm (samples from periodontitis patients based on probing depths). Sites with PD greater than 10 mm were excluded due to low occurrence (≤1%) and sampling difficulty. Full-mouth periodontal examinations were conducted by two calibrated practitioners; interpersonal calibration was performed on five patients who were not recruited in this study. The consistency of the measurements based on PD was calculated at the site level [23]. The results for 840 exploration sites demonstrated a consistency rate of 97.5%, with a Kappa value of 0.932. Individual calibration was performed on another five patients who were not recruited in this study, and the intraclass correlation coefficients for PD were 96% and 95% within 1 mm.

### 2.2. Sample Collection and Processing

Subgingival plaque samples were collected one week after full-mouth periodontal examination and supragingival scaling [23]. All participants were requested to refrain from food for 2 h and oral hygiene for 12 h before sampling. Samples were collected after isolating the selected sampling area with cotton rolls and air drying gently, supragingival plaque was removed carefully with curettes and cotton rolls, and subgingival samples were obtained by placing a sterile Gracey curette from the apical extent of the periodontal pocket or gingival crevice and drawing it coronally with slight pressure [18]. For health subjects, one subgingival plaque sample was obtained from six sites of first molars, premolars, and central incisors without BOP. For each periodontitis patient, three separate subgingival plaque samples were collected from PD 0–3 mm, PD 4–5 mm, and PD 6–9 mm sites. Collected samples were immediately placed in 1× PBS buffer on ice and transported to the laboratory within 2 h. The samples were centrifuged at 10,000× *g* for 10 min, and the pellet was washed with 1× PBS buffer and then stored at −80 °C until DNA extraction and sequencing.

### 2.3. DNA Extraction, PCR Amplification, and Sequencing

Total DNA was extracted from 64 samples using the FastPure Stool DNA Isolation Kit (MJYH, Shanghai, China) according to manufacturer’ s instructions. The quality and concentration of DNA were determined by 1.0% agarose gel electrophoresis and a NanoDrop^®^ ND-2000 spectrophotometer (Thermo Scientific Inc., Waltham, MA, USA). The hypervariable region V3-V4 of the bacterial 16S rRNA gene was amplified with primer pairs 27F (5′-AGAGTTTGATCCTGGCTCAG-3′) and 533R (5′-TTACCGCGGCTGCTGGCAC-3′) [24] by an ABI GeneAmp^®^ 9700 (Thermo Scientific Inc., Waltham, MA, USA). PCR amplification was performed under the following conditions: initial denaturation at 95 °C for 3 min followed by 27 cycles of denaturing at 95 °C for 30 s, annealing at 55 °C for 30 s, extension at 72 °C for 45 s, single extension at 72 °C for 10 min, followed by a holding step at 4 °C. All samples were amplified in triplicate. The PCR product was extracted from 2% agarose gel and purified and then quantified using Synergy HTX (Biotek, Winooski, VT, USA). Purified amplicons were pooled in equimolar amounts and paired-end sequenced on an Illumina NextSeq 2000 PE300 platform (Illumina, San Diego, CA, USA) following the standard protocols by Majorbio Bio-Pharm Technology Co., Ltd. (Shanghai, China).

### 2.4. Bioinformatic Analysis, Statistical Analysis, and Visualization

Raw FASTQ files were de-multiplexed using an in-house perl script and then quality-filtered by fastp (version 0.19.6) [25] and merged by FLASH (version 1.2.11) [26] with the following criteria: the PE300 reads were truncated at any site receiving an average quality score of <20 over a 50 bp sliding window, and the truncated reads shorter than 50 bp were discarded; reads containing ambiguous characters were also discarded; only overlapping sequences longer than 10 bp were assembled according to their overlapped sequence; the maximum mismatch ratio of overlap region is 0.2; reads that could not be assembled were discarded; samples were distinguished by their barcode and primer sequences, with exact matching required for the barcode and allowing up to two nucleotide mismatches in primer sequences. Then, the optimized sequences were clustered into operational taxonomic units (OTUs) using Usearch (version 11) [27] with a 97% sequence similarity level. The most abundant sequence for each OTU was selected as a representative sequence. To minimize the effects of sequencing depth on alpha and beta diversity measures, the number of 16S rRNA gene sequences from each sample was rarefied to 20,000, which still yielded an average Good’s coverage of 99.09%. The taxonomy of each OTU representative sequence was analyzed by RDP Classifier (version 2.13) [28] against the eHOMD database (version 15.3) using a confidence threshold of 97%.

The comparison of alpha diversity and microbiota composition at phylum and genus level among groups was calculated by the Kruskal–Wallis test with FDR (false discovery rate), and post hoc comparisons were performed using Welch’s (uncorrected) tests. Beta diversity was assessed using principal coordinates analysis (PCoA) based on Bray–Curtis and Abund-Jaccard distances, with statistical significance determined by the Wilcoxon rank-sum test with FDR adjustment. Co-occurrence network correlations were determined using Spearman’s correlation analysis in RStudio (version 2022.07.2, R version 4.2.1) and visualized using Cytoscape (version 3.8.0).

## 3. Results

**Figure 1:**
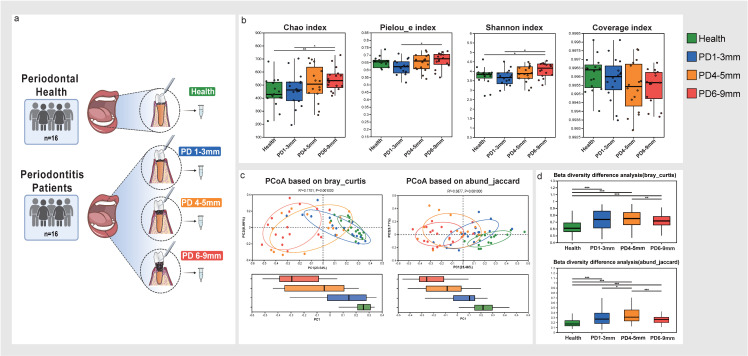
The flowchart of subgingival plaque sample collection and comparison of microbiota alpha and beta diversity among the four groups. (**a**) The sampling strategies of this study. Subgingival plaque samples collected from periodontal healthy subjects were designated as the health group; subgingival plaque samples collected from periodontitis patients from a probing depth of 0–3 mm, 4–5 mm, and 6–9 mm sites were designated as the PD 0–3 mm group, PD 4–5 mm group, and PD 6–9 mm group, respectively. (**b**) Microbial alpha diversity as calculated by the Chao, Pielou_e, Shannon, and Coverage index. Comparison among groups were calculated by the Kruskal–Wallis test with FDR (false discovery rate); Welch’s (uncorrected) test was used as a post hoc test. (**c**) The principal coordinates analysis (PCoA) of beta diversity among the four groups was based on the Bray–Curtis and Abund-Jaccard tests. The ellipses indicate 95% confidence intervals for sample groups. (**d**) The beta diversity difference analysis based on the Bray–Curtis and Abund-Jaccard tests were calculated by the Wilcoxon rank-sum test with FDR. The y-axis represents the beta diversity distance values. * *p* ≤ 0.05; ** *p* ≤ 0.01; *** *p* ≤ 0.001.

Sixteen periodontal healthy subjects and sixteen patients with stage III periodon-titis were enrolled, with the demographic and clinical characteristics of the participants shown in Table 1. The average age of the healthy subjects was 29.7 ± 5.5 years, while the periodontitis patients had an average age of 41.7 ± 12.7 years. The periodontitis patients exhibited higher BOP (83.42 ± 0.5%) compared to the health group (4.53 ± 2.77%). Subgingival plaque samples were collected from one site in each healthy subject and from three sites at PDs of 0–3 mm, 4–5 mm, and 6–9 mm in each periodontitis patient, resulting in a total of 64 subgingival plaque samples, grouped as follows: health group, PD 0–3 mm group, PD 4–5 mm group, and PD 6–9 mm group (Figure 1a, Table 1). Following quality control, 3,385,680 optimized reads were retained, with an average of 52,901 reads per sample. At a similarity level of 97%, a total of 3,454 operational tax-onomic units (OTUs) were identified, belonging to 14 phyla, 31 classes, 55 orders, 103 families, 200 genera, and 520 species.

### 3.1. Microbial Diversity Analysis in Health and Periodontitis Across Varying Probing Depths

Microbial alpha diversity was calculated using the Chao, Pielou_e, Shannon, and Coverage indices (Figure 1b). The Chao index, which evaluates the total number of taxa within the group, revealed that the PD 6–9 mm group had significantly higher richness compared to the PD 0–3 mm group and the health group. The Pielou_e index, which measures microbiota evenness, showed significantly higher microbial evenness in the PD 6–9 mm group compared to the PD 0–3 mm group. The Shannon index, which reflects overall diversity, also indicated that the PD 6–9 mm group had higher microbial alpha diversity than both the PD 0–3 mm group and the health group. The coverage index, which represents sequencing coverage, showed that the Good’s coverage among the four groups had no significant differences. These results indicate that the subgingival microbial richness and diversity in the PD 6–9 mm sites of periodontitis patients are greater than that in healthy subjects. Additionally, microbial richness, evenness, and diversity in the PD 6–9 mm sites of periodontitis patients were higher than in the PD 0–3 mm sites.

To further elucidate the microbial structure, principal coordinates analyses (PCoA) were performed based on the OTU data (Figure 1c,d). Beta diversity analysis using Bray-Curtis and Abund-Jaccard indices revealed that the health group exhibited significantly lower beta diversity compared to all periodontitis groups. The PD 6–9 mm group had significantly lower beta diversity than the PD 4–5 mm group. Moreover, the Abund-Jaccard analysis showed that the PD 0–3 mm group had significantly lower diversity than the PD 4–5 mm group. These results suggest that the subgingival microbial structure in periodontitis patients differs from that in healthy subjects, with microbial community distances increased from PD 0–3 mm to PD 4–5 mm and then decreased from PD 4–5 mm to PD 6–9 mm.

### 3.2. Compositions of Subgingival Microbiota at the Phylum Level

As for taxonomic analysis at the phylum level, the four groups were all dominated by Firmicutes (relative abundance ranged from 26.99 to 33.20%) and were abundant in Bacteroidetes (from 16.86 to 22.65%), Fusobacteria (from 13.69 to 15.26%), Proteobacteria (from 5.99 to 21.21%), Actinobacteria (from 9.21 to 13.09%), and Saccharibacteria (TM7) (from 2.04 to 7.38%) at the phylum level (Figure S1).

**Figure 2:**
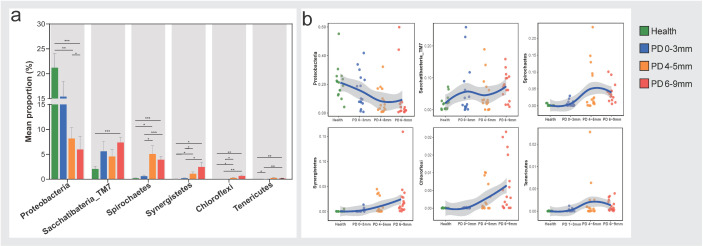
Phylum-level microbiota composition has significant relative abundance differences among the four groups. (**a**) Six phyla, including Proteobacteria, Saccharibacteria (TM7), Spirochaetes, Synergistetes, Chloroflexi, and Tenericutes, showed significant differences among the four groups. The Kruskal–Wallis test with FDR was used to compare differences among groups; Welch’s (uncorrected) test was used as a post hoc test. * *p* < 0.05, ** *p* < 0.01, *** *p* < 0.001. (**b**) Relative abundance shift patterns for each of the six phyla across the four groups. Each point in the figure shows the relative abundance of the bacterial phylum in the sample. The blue trend line is the fitted curve of the average relative abundance, and the gray shaded area shows the interval of the standard deviation.

A total of six phyla with significant differences among the four groups were iden-tified, including Proteobacteria, Saccharibacteria (TM7), Spirochaetes, Synergistetes, Chloroflexi, and Tenericutes (Figure 2a). Moreover, relative abundance shift patterns of each phylum across the four groups are shown in separate figures (Figure 2b). The Proteobacteria exhibited a decreasing trend from the health to disease states, with the health group showing significantly higher relative abundance compared to the PD 4–5 mm and PD 6–9 mm groups. In contrast, the relative abundance of the other five phyla, Saccharibacteria (TM7), Spirochaetes, Synergistetes, Chloroflexi, and Tenericutes, ex-hibited increasing trends from the health to disease states.

### 3.3. Compositions of Subgingival Microbiota at the Genus Level

**Figure 3:**
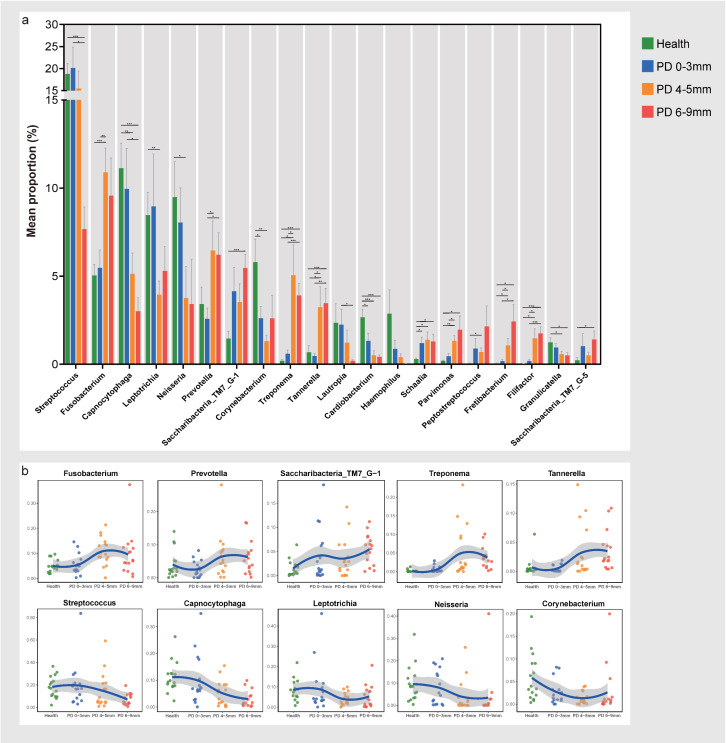
Genus-level microbiota composition has significantly different relative abundance among the four groups. (**a**) The top 20 genera have significant differences among the four groups. The Kruskal–Wallis test with FDR was used to compare differences among groups, and Welch’s (uncorrected) test was used as a post hoc test. * *p* < 0.05, ** *p* < 0.01, *** *p* < 0.001. (**b**) Relative abundance shift patterns for the top 10 genera across the four groups.Each point in the figure shows the relative abundance of the bacterial phylum in the sample. The blue trend line is the fitted curve of the average relative abundance, and the gray shaded area shows the interval of the standard deviation.

To further elucidate the differences in microbial composition, we conducted a comparative analysis of relative abundance at the genus level. The top 20 genera that had significant differences among the four groups included Streptococcus, Fusobacte-rium, Capnocytophaga, Leptotrichia, Neisseria, Prevotella, Saccharibacteria (TM7) [G-1], Corynebacterium, Treponema, Tannerella, Lautropia, Cardiobacterium, Hae-mophilus, Schaalia, Parvimonas, Peptostreptococcus, Fretibacterium, Filifactor, Gran-ulicatella, and Saccharibacteria (TM7) [G-5] (Figure 3a). Shift patterns in relative abundance for the top 10 genera are shown in separate figures (Figure 3b). Among these, the relative abundance of Fusobacterium, Prevotella, Saccharibacteria (TM7) [G-1], Treponema, and Tannerella increased from the health to periodontitis states, indicating an elevated composition percentage in the periodontal inflammation sites. In contrast, Streptococcus, Capnocytophaga, Leptotrichia, Neisseria, and Corynebacte-rium exhibited decreasing trends from the health group to periodontitis. Moreover, Corynebacterium, Cardiobacterium, and Schaalia showed significant differences be-tween the health and PD 0–3 mm groups.

### 3.4. Co-Occurrence Network Based on Species Level Revealed Different Interaction Patterns in Each Group

**Figure 4:**
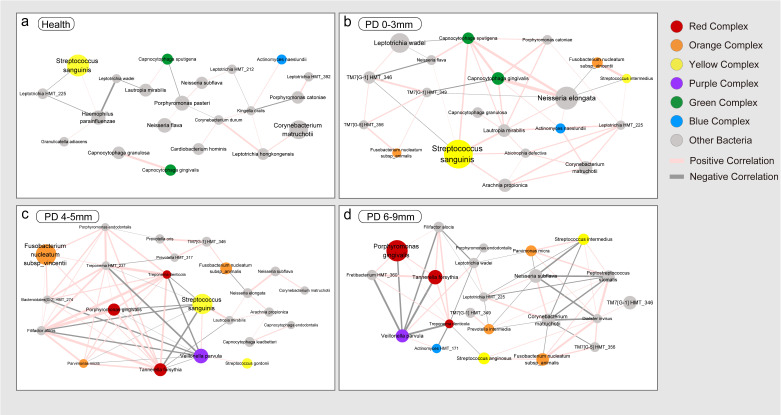
Co-occurrence network of species with relative abundance > 1% in each group. (**a**) Health group. (**b**) PD 0–3 mm group. (**c**) PD 4–5 mm group. (**d**) PD 6–9 mm group. Species’ relative abundance and correlation coefficient values are presented by nodes and edges. The size of the nodes and node fonts represent the average relative abundance of species in each group. The color of the nodes (red, orange, yellow, purple, green, and blue) represents the six periodontitis complexes according to the Socransky classification with species not belonging to the six complexes shown as grey nodes. The correlation coefficient r values were determined using Spearman correlation analysis, with the thickness of the lines positively correlated with the absolute value of the r values. Pink edges indicate positive correlations (r > 0.4, *p* < 0.05), while grey edges indicate negative correlations (r < −0.4, *p* < 0.05).

Monofactor network analysis is widely used to infer the taxon–taxon interactions in bioinformatic pipelines [29]. In this study, the number of species with a relative abundance > 1% in the health, PD 0–3 mm, PD 4–5 mm, and PD 6–9 mm groups was 24, 24, 24, and 23, respectively (all the species are listed in Table S1). Co-occurrence network analysis was carried out to investigate how those microbial taxa function together using Spearman correlation analysis (Figure 4). The names and mean relative abundances of all detected bacterial taxa are presented in Table S2.

The health group had one yellow complex species (*Streptococcus sanguinis*), two green complex species (*Capnocytophaga gingivalis* and *Capnocytophaga sputigena*), and one blue complex species (*Actinomyces naeslundii*). Those species were also present in the PD 0–3 mm group; moreover, the orange complex species (*Fusobacterium nucleatum* subsp. *animalis* and *Fusobacterium nucleatum* subsp. *vincentii*) began to appear in the PD 0–3 mm group. When it comes to diseased sites, the three red complex species (*Porphyromonas gingivalis*, *Tannerella forsythia*, and *Treponema denticola*), with positive correlations among each other, appeared both in the PD 4–5 mm group and the PD 6–9 mm group. In the PD 6–9 mm group, apart from Socransky’s complex, *Filifactor alocis* and *Fretibacterium HMT_360* constructed positive interactions with the three red complex species. Morever, two species belonging to the orange complex (*Fusobacterium nucleatum* subsp. *animalis* and *Parvimonas micra*) and one species belonging to the purple complex (*Veillonella parvula*) were present in the PD 4–5 mm group and PD 6–9 mm group.

Regarding correlation dynamics, in the health group had a total of 22 correlations, of which 14 (63.64%) were positive, and 8 (36.36%) were negative correlations. In the PD 0–3 mm group, the correlations increased to 45, of which 37 (82.22%) were positive, and 8 (17.78%) were negative correlations. In the PD 4–5 mm group, there were a total of 60 correlations, of which 43 (71.67%) were positive, and 17 (28.33%) were negative correlations. In the PD 6–9 mm group, there were a total of 56 correlations, of which 30 (53.57%) were positive, and 26 (46.43%) were negative correlations. These results suggest that the correlation relationships within the subgingival plaque microbiota are simpler in the health group, while more complex interactions are present in periodontitis patients, regardless of whether the sites are healthy or diseased. This indicates more intense cooperative or antagonistic relationships among bacterial species in periodontitis.

### 3.5. Core and Unique Microbial Species Across Health and Periodontitis

**Figure 5:**
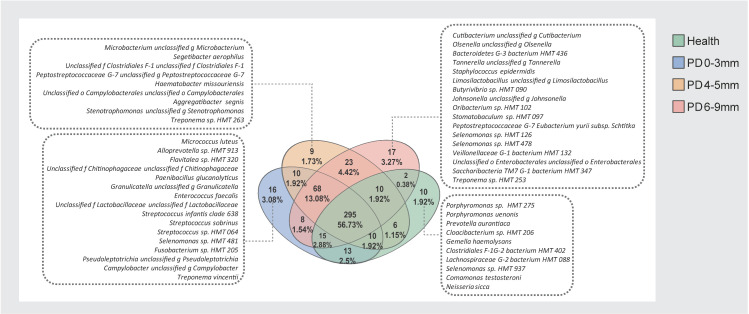
Venn diagrams of the four groups based on the species level. The numbers and percentages in the overlapping areas represent species shared between corresponding groups. Species unique to each group are listed in the surrounding text boxes.

A Venn diagram was used to show the number of species common to or unique among the four groups. In the health, PD 0–3 mm, PD 4–5 mm, and PD 6–9 mm groups, 361, 435, 431, and 438 species were detected, respectively (Figure 5). The number of unique species in each group was 10, 16, 9, and 17, respectively. Among all species detected, 295 species (56.73%) were shared by all four groups, representing the core microbiome of subgingival dental plaque across both health and disease states. Additionally, 68 species were shared by the PD 0–3 mm, PD 4–5 mm, and PD 6–9 mm groups, suggesting they may represent a common microbiome for periodontitis patients. A total of 23 species were shared exclusively by the PD 4–5 mm and PD 6–9 mm groups, which may preferentially inhabit periodontal pockets. Finally, 13 species were shared only between the health and PD 0–3 mm groups. The names of these species are listed in Table S3.

## 4. Discussion

Previous studies exploring probing depth-related differences in the subgingival microbiota have faced several limitations, including reliance on integrated data rea-nalysis [17], use of paper point sampling that primarily captures superficial plaque layers [20], and small sample sizes [18,19]. To address these gaps, we conducted this study to investigate the subgingival microbiota composition across different probing depth sites in stage III periodontitis patients using the curette method, which enables the collection of inner-layer plaque. By comparing these profiles to those from healthy subjects, we provide a more detailed understanding of microbial shifts associated with periodontal pocket depth, moving beyond the conventional health versus disease framework [7,9].

The PD 6–9 mm group exhibited significantly higher microbial richness than both healthy subjects and the PD 0–3 mm group, and higher evenness compared to the PD 0–3 mm group. These findings suggest that as periodontal pockets deepen, they may cre-ate more diverse and anaerobic-friendly niches that support a broader and more bal-anced microbial community. While some earlier studies reported similar trends, others found either partial or conflicting patterns [18,19,20]. For instance, Paula et al. [19] noted higher evenness in chronic periodontitis but did not observe differences in richness across depth categories, and Shi et al. [18] found increased richness in periodontitis overall. Our observation of a steady increase in alpha diversity with pocket depth contrasts with He et al. [20], who reported a non-linear pattern, which may be attributable to differences in sampling methodology. Regarding beta diversity, our re-sults confirm that subgingival microbial community structure differs significantly be-tween health and periodontitis, consistent with previous findings [7,9,20]. We also ob-served depth-associated compositional shifts across multiple genera, most of which were consistent with previous reports [17,19,20]. Notably, the PD 0–3 mm group in our study showed a distinct community structure compared to the health group. This differs from some earlier reports, which suggested minimal differences between healthy sites and shallow pockets in periodontitis [19,20]. The divergence may reflect subtle ecological shifts in clinically “healthy” sites within periodontitis patients—shifts that could indicate early microbial dysbiosis. Supporting this, we observed significant changes in the relative abundance of Corynebacterium, Cardiobacterium, and Schaalia between healthy subjects and the PD 0–3 mm group. These genera may serve as po-tential early indicators of subclinical disease or microbial instability preceding overt periodontitis.

Consistent with the previous study [20], our co-occurrence network analysis re-vealed greater interaction complexity in moderate and deep periodontal pockets compared to shallow sites and healthy controls. Unlike previous studies that relied on genus-level ASVs, we performed species-level analysis, enabling more precise identi-fication of key microbial players. Moreover, by incorporating Socransky’s complex [11], we were able to contextualize taxon–taxon interactions within established pathogenic frameworks, providing clearer insights into microbial succession and disease progres-sion. Notably, we found that red complex species Porphyromonas gingivalis, Tannerella forsythia, and Treponema denticola only presented in the PD 4–5 mm and 6–9 mm sites of periodontitis and had positive correlations with each other. In the PD 6–9 mm group, Filifactor alocis, Fretibacterium HMT_360, and the three red complex species constructed positive interactions with each other, indicating their importance in periodontitis pathogenesis. Filifactor alocis is a newly confirmed periodontitis pathogen [30], with comparative resistance to oxidative stress, production of unique proteases and colla-genases that can cause structural damage to host cells, and dysregulation of the im-mune system. There also have been studies [10,31] that support the potential of Freti-bacterium HMT_360 as a periodontitis pathogen. In contrast, orange complex species appeared earlier than red complex species; Fusobacterium nucleatum subsp. animalis and vincentii both showed in the PD 0–3 mm site of the periodontitis group. Studies indi-cated that they serve as bridge bacteria for early colonizers and late-colonizing patho-gens [32] and are considered to be the precedents of the red complex for colonization and proliferation [12].

The PD 6–9 mm group showed significantly higher relative abundance of the phylum Saccharibacteria (TM7) than the health group. Furthermore, at the genus level, the Saccharibacteria (TM7) [G-1] and Saccharibacteria (TM7) [G-5] also showed significantly higher relative abundance in the PD 6–9 mm group than the health group. Those findings are consistent with previous studies, and the association of TM7 with periodontitis has long been suggested [17,33,34]. Pauline et al. [34] observed that diseased sites (PD ≥ 5 mm) were enriched in TM7 compared to healthy sites (PD ≤ 3 mm) in the same oral cavity. Three species (TM7 [G-1] HMT_346, TM7 [G-1] HMT_349, and TM7 [G-5] HMT_356) belonging to the TM7 phylum have positive correlations with the orange complex bacteria *Fusobacterium nucleatum* subsp. *animalis* in the three periodontitis groups. Interestingly, a previous study [7] reported the core subgingival microbiome in health and periodontitis, and the above mentioned three TM7 species were included in the periodontitis core subgingival microbiome and were the only three species in the core microbiome belonging to the TM7 phylum. Moreover, TM7 [G-1] HMT_349 has a positive interaction with another orange bacteria *Prevotella intermedia* in the PD 6–9 mm group. Socransky’s orange complex is considered the precedent of the red complex for colonization and proliferation [12]. Saccharibacteria (formerly known as TM7) are a group of ultrasmall bacteria that are recalcitrant to cultivation due to their epibiotic parasitic lifestyle and belong to the Candidate Phyla Radiation [35]. The recently successfully isolated TM7 representatives from the human oral cavity shed light on the role of TM7 in periodontitis in vitro [36,37].

The subgingival plaque sampling mainly relies on two methods: the curette method and the adsorption method using paper points [38]; both of them were widely used. A study carried out by Liu et al. [21] collected subgingival plaques sequentially using filter paper and curettes, and the 16S rRNA gene sequencing results showed no significant differences between the operational taxonomic unit numbers, but the composition of some bacteria was significantly different between the two groups. A recently published study [20] using the adsorption method to study health, stage I/II, and stage III/IV periodontitis with different probing depth showed that alpha diversity initially increased and then decreased. This is controversial with respect to our study which showed the the PD 6–9 mm group has the highest alpha diversity, and the differences may be influenced by the sampling methods. Furthermore, the microbial structure, composition, and network analysis have the same trend between the previous study [20] and our study. The combination of the curette and adsorption methods could be considered to study the subgingival microbiome comprehensively in the future. Furthermore, with the development of imaging technology [32], sequencing combined with imaging could serve as a robust tool to reveal the composition and biogeography of subgingival plaques.

## 5. Conclusions

This study demonstrated the subgingival microbial diversity, structure, composi-tion, and interspecies interactions varied significantly across health sites and perio-dontal pockets of different depths (PD 0–3 mm, PD 4–5 mm, and PD 6–9 mm) in stage III periodontitis. Several genera exhibited notable shifts in relative abundance from health to disease, with Corynebacterium and Cardiobacterium significantly decreased and Schaalia increased in shallow sites of periodontitis compared to health, potentially indicating early microbial alternations. Moderate periodontal pockets (PD 4–5 mm) exhibited the most complex co-occurrence networks, followed by deep and shallow periodontal pockets. In deep periodontal pockets, Filifactor alocis, Fretibacterium HMT_360, and the red complex species (Porphyromonas gingivalis, Tannerella forsythia, and Treponema denticola) constructed positive associations with each other, supporting their cooperative role in advanced periodontitis pathogenesis. Together, these findings underscore the value of probing depth-based microbiome analysis in understanding microbial succession and interactions during disease progression and may contribute to identifying early microbial markers and potential intervention targets in periodontitis.

## Figures and Tables

**Table 1:** Demographic information and clinical characteristics of the thirty-two individuals.

		Health		Periodontitis	
Number		16		16	
Gender	Male	8		9	
	Female	8		7	
Age		29.73 ± 5.51		41.69 ± 12.68	
Number of teeth		28		26.94 ± 1.18	
Probing depth (PD)		≤3 mm		4.02 ± 1.87	
Percentage of PD		PD 0–3 mm	100%	PD 0–3 mm	55%
				PD 4–5 mm	25%
				PD 6–9 mm	19%
				PD ≥ 10 mm	1%
Bleeding on probing		4.53 ± 2.77%		83.42 ± 0.5%	

Values shown as the means ± standard deviations.

## Data Availability

The raw sequencing reads were deposited into the NCBI Sequence Read Archive (SRA) database (Accession Number: PRJNA1188528), (https://www.ncbi.nlm.nih.gov/bioproject/?term=(PRJNA1188528)%20AND%20bioproject_sra[filter]%20NOT%20bioproject_gap[filter], accessed on 21 November 2024).

## Supplementary Materials

The following supporting information can be downloaded at https://www.mdpi.com/article/10.3390/microorganisms13040930/s1, Figure S1: Taxonomy at phylum level; Table S1: Number of species with relative abundance > 1%; Table S2: Mean relative abundance of detected bacteria; Table S3: Number of species shared among four groups.
