# Supplementary figures and images for: The Subgingival Microbial Composition in Health and Periodontitis with Different Probing Depths
Source: Microorganisms. 2025 Apr 17;13(4):930. doi: 10.3390/microorganisms13040930 (PMC12029188; doi:10.3390/microorganisms13040930)

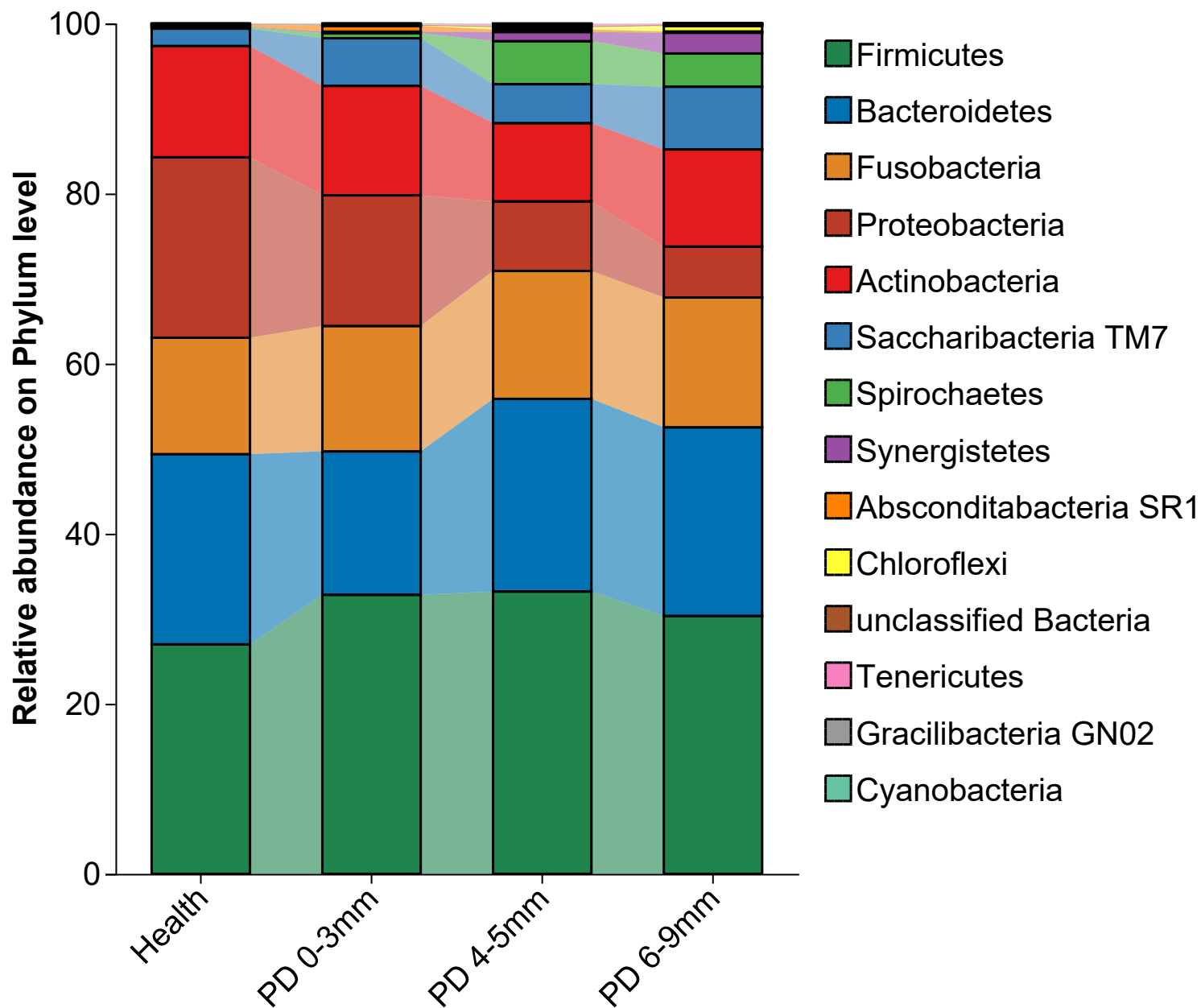

Supplement: Supplementary file 1 [file microorganisms-13-00930-s001.zip › Figure S1. Taxonomy at phylum level.pdf]
